# Long-term follow-up and comparison of programmable and non-programmable ventricular cerebrospinal fluid shunts among adult patients with different hydrocephalus etiologies: a retrospective cohort study

**DOI:** 10.1007/s00701-023-05734-z

**Published:** 2023-08-09

**Authors:** Kuan-Hung Chen, Peng-Wei Hsu, Bo-Chang Wu, Po-Hsun Tu, Yu-Chi Wang, Cheng-Chi Lee, Yin-Cheng Huang, Ching-Chang Chen, Chi-Cheng Chuang, Zhuo-Hao Liu

**Affiliations:** 1 Department of Neurosurgery, Chang Gung Memorial Hospital at Linkou, Chang Gung Medical College and University, Taoyuan, Taiwan; 2 Department of Surgery, Johns Hopkins University School of Medicine, Baltimore, MD USA; 3 School of Medicine, National Tsing Hua University, Hsinchu, Taiwan

**Keywords:** Adult hydrocephalus, Cerebrospinal fluid shunt, Programmable valve, Shunt revision, Long-term outcome

## Abstract

**Background:**

Programmable valve (PV) has been shown as a solution to the high revision rate in pediatric hydrocephalus patients, but it remains controversial among adults. This study is to compare the overall revision rate, revision cause, and revision-free survival between PV and non-programmable valve (NPV) in adult patients with different hydrocephalus etiologies.

**Method:**

We reviewed the chart of all patients with hydrocephalus receiving index ventricular cerebrospinal fluid (CSF) shunt operations conducted at a single institution from January 2017 to December 2017. Patients included in the study were followed up for at least 5 years. Statistical tests including independent *t*-test, chi-square test, and Fisher’s exact test were used for comparative analysis, and Kaplan-Meier curve using log-rank test was performed to compare the revision-free survival between the PV and NPV groups.

**Results:**

A total of 325 patients were included in the study, of which 181 patients were receiving PVs and 144 patients receiving NPV. There were 23 patients (12.8%) with PV and 22 patients (15.3%) with NPV receiving initial revision. No significant statistical difference in the initial revision rate was observed between the two groups (*p* = 0.52). No survival difference was found between the PV and NPV groups. However, better revision-free survival was noted in the PV group among idiopathic normal pressure hydrocephalus (iNPH) (*p* = 0.0274) and post-traumatic hydrocephalus (*p* = 0.017).

**Conclusions:**

The combination of the different etiologies of hydrocephalus and the features of PV and NPV results in different outcomes—revision rate and revision-free survival. PV use might be superior to NPV in iNPH and post-traumatic hydrocephalus patients. Further studies are needed to clarify the indications of PV use in adult hydrocephalus patients.

## Background

Hydrocephalus, which refers to the abnormal accumulation of cerebrospinal fluid (CSF) within the cerebral ventricle, is a common neurosurgical disorder that each neurosurgeon would encounter in daily practice. The estimated prevalence of hydrocephalus is 1 to 1.5% in general population [12]. The pathophysiology of hydrocephalus formation comprises CSF overproduction, CSF malabsorption, or blockage in the CSF circulatory system that can result from nontraumatic intracerebral hemorrhage, intracranial neoplasm, post-traumatic hydrocephalus, idiopathic normal pressure hydrocephalus, or ischemic stroke [11]. Hydrocephalus is one of the treatable causes of dementia [18], and CSF diversion operation can resolve hydrocephalus and ultimately improve patient’s cognitive function. Ventriculo-peritoneal shunt and ventriculo-atrial shunt are the most common types of CSF diversion operation performed worldwide [38].

Although ventricular CSF shunt, a frequently performed neurosurgical operation, has been developed for 70 years [37], the high shunt failure rate is a problem that remains to be solved. Several studies have reported rates of CSF shunt failure, which were as high as 32% from 1990 to 2009 in the USA, as reported by Reddy et al*.* [31]. Rocio et al. revealed a shunt revision rate of 17.4% within the first year of surgery from 2003 to 2014 in the UK and Ireland [11]. Nadia et al. reported a shunt revision rate of 20.7% from 2008 to 2017 in Norway [24]. Infection and obstruction caused by blood clots or fibrin formation are the most common causes of shunt malfunction or failure [24]. Other causes, such as valve malfunction, lead to under-drainage or over-drainage and technical issues.

Several attempts have been made to reduce the need for revision, and the programmable valve (PV) has been proposed as a solution to the high revision rate of the traditional non-programmable valve (NPV) for its adjustability. However, the higher cost of PVs raises the concern that the efficacy and durability are really better in PVs than in NPVs in real-world clinical practice. A meta-analysis in 2017 by Li et al. [22] included 3 randomized-controlled trials and 8 observational studies, comparing the efficacy and safety between PVs and NPVs [2, 7, 10, 14, 19, 21, 23, 25, 26, 34, 39]. It noted that PVs could reduce the revision rate and over- or under-drainage complication rates in pediatric patients compared to traditional NPVs. However, for adult patients, PVs were not superior to NPVs in terms of risk for shunt revision, overall complication, infection, and over- or under-drainage. Agarwal et al. found a similar revision rate around 20% in between PVs and NPVs in adults among various hydrocephalus etiologies [1]. However, Lorenzo et al. in 2020 revealed that PVs led to the reduced revision rate in adult patients with idiopathic normal pressure hydrocephalus compared to NPVs [33]. Orrego et al. and Darkwah et al. showed that adult nontraumatic subarachnoid hemorrhage patients with PVs had lower revision rates and over- or under-drainage rates [9, 28]. No widely accepted clinical practice guideline regarding the indication of PV in adult patients was available due to the conflicting results from several previous studies [9].

The discrepancy among several previous studies might be ascribed to the different causes of hydrocephalus and the relatively short follow-up intervals. To the best of our knowledge, no previous studies have compared the efficacy and revision rate between PVs and NPVs in adult patients with different hydrocephalus etiologies for a follow-up period of time at least 5 years. Our study is designed to compare the overall revision rate, revision cause, and revision-free survival between PVs and NPVs, and subgroup analysis among different hydrocephalus etiologies to identify the population who may benefit from either PVs or NPVs.

## Methods

### Study design and patient enrollment

**Fig. 1:**
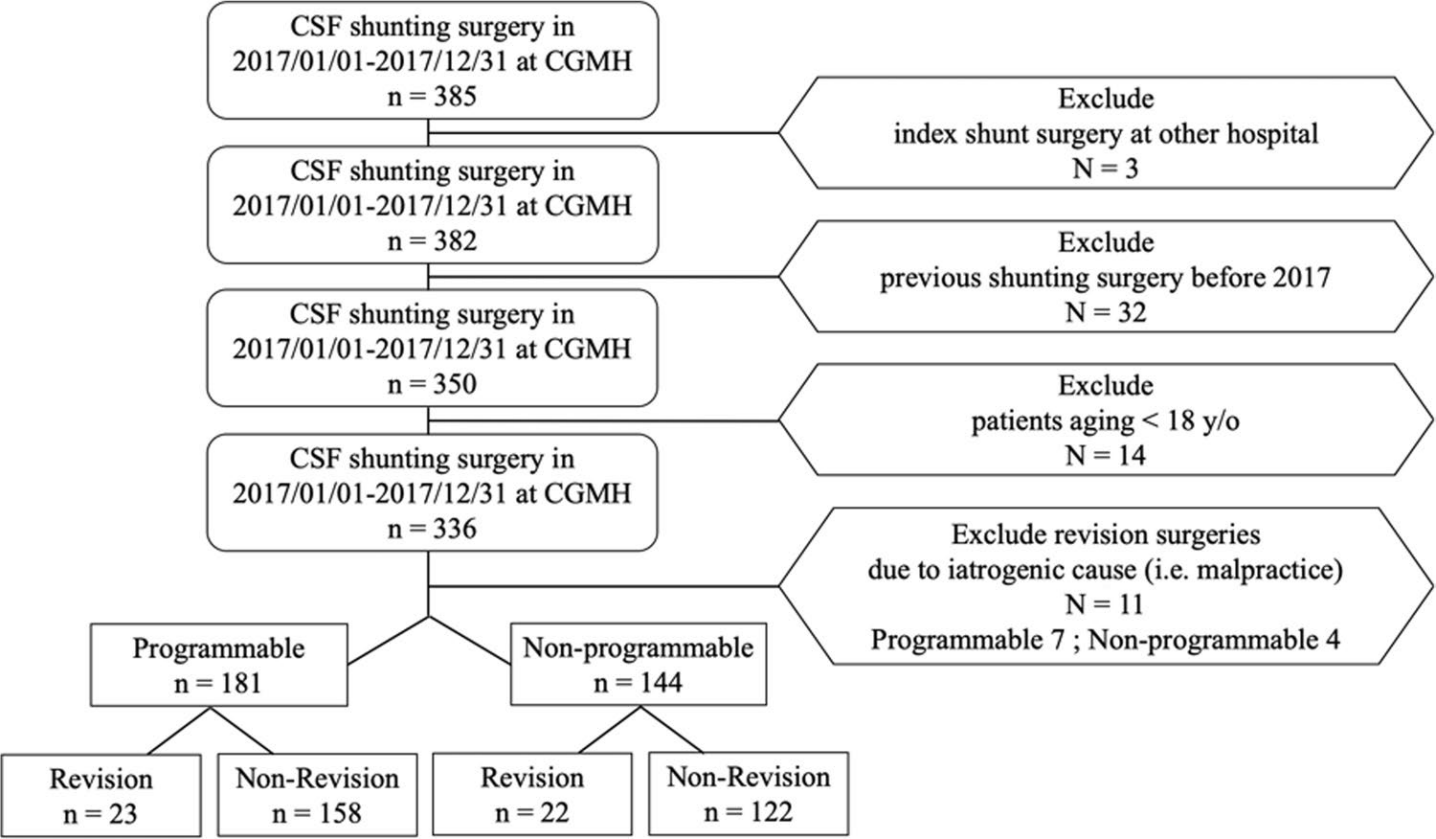
Patient enrollment flow chart

We reviewed the chart of all patients with hydrocephalus receiving index ventricular CSF shunt operations conducted at a single institution from January 2017 to December 2017. The study is registered retrospectively on ClinicalTrials.gov (ClinicalTrials.gov Identifier: NCT05534659). The study was designed and reported in line with the STROBE guideline [36]. The index ventricular CSF shunt was defined as the first ventricular CSF shunt procedure in a series of shunt procedures. Patients who received index ventricular CSF shunt at other hospitals, who received index ventricular CSF shunt before January 1st, 2017, who were younger than 18, and who had had ventricular CSF revision operations were all excluded. Figure 1 is a flow chart illustrating patient enrollment. All patients included in the study were followed up for at least 5 years until June 30th 2022. All CSF shunt operations were performed by the board-certified neurosurgeons or the neurosurgery senior residents under supervision. The choice between programmable and non-programmable CSF shunt valves was based on attending neurosurgeons’ preferences in our institution. Three different programmable shunts (Medtronic Strata, B-Braun ProGav, Codman Certas) and one non-programmable shunt (Medtronic CSF-flow control valve) were used in our institute. Non-programmable shunts used in this study had two different operating pressure valves: low with 3 cm-H_2_O and medium with 8.5 cm-H_2_O for CSF flow rate of 5 mL/h (Medtronic CSF-flow control valve) [8].

There were a total of 11 patients excluded from our study due to iatrogenic cause. In PV groups, 7 patients received revision operation with 3 ventricular tip malposition, 2 ventriculostomy tract hematoma, 1 distal tip malposition, and 1 tube exposure due to inadequate tunneling. In NPV groups, 4 patients received revision operation with 1 ventricular tip malposition, 1 ventriculostomy tract hematoma, 1 distal tip malposition, and 1 tube exposure due to inadequate tunneling. These patients were excluded to focus on the interplay between patient’s hydrocephalus etiology and the features of PV/NPV.

### Data collection

The study was ethically approved by the institutional review board (IRB number: 202200775B0) with the permission of the waiver of informed consent form after de-linking the patient’s identity and collected health information. All required data were obtained from the electronic medical record system at a single institute. Patient’s demographic data were collected, including age, height, body weight, body mass index (BMI), and medical comorbidities (i.e., hypertension, diabetes mellitus, and hyperlipidemia). A ventricular CSF shunt operation was indicated for different etiologies of hydrocephalus-nontraumatic intracranial hemorrhage, including cerebral aneurysmal subarachnoid hemorrhage (aSAH), hypertensive intracerebral hemorrhage (ICH), arteriovenous malformation (AVM), spontaneous intraventricular hemorrhage, intracranial neoplasm (including primary brain tumor, malignant metastatic brain tumor, and leptomeningeal seeding), idiopathic normal pressure hydrocephalus (iNPH), traumatic brain injury, ischemic stroke, central nervous system infection, and others (such as dural arteriovenous fistula, Wilson’s disease with CNS involvement and idiopathic intracranial hypertension). Normal pressure hydrocephalus patients included in our study were all iNPH patients without identifiable risk factors, whose diagnosis meets the diagnostic criteria as below: age more than or equal to 60 years, abnormal ventriculomegaly shown on brain cranial computed tomography or magnetic-resonance image or at least one symptom of iNPH triad: incontinence, gait disturbance, or dementia [17]. Several perioperative factors such as operation duration, ventricular catheter laterality, distal shunting site, previous external ventricular drainage (EVD) or not, and postoperative antibiotic use duration were recorded.

### Outcome measures

Primary outcomes of the study included the cause of shunt revision, shunt revision rate, type of the revision operation, and revision-free survival between PVs and NPVs. The causes of ventricular CSF shunt revision were divided into four categories: (1) valve function-related, (2) CSF shunt system obstruction, (3) infection, and (4) technical skill-related (i.e., ventricular/distal tip malposition, postoperative ventriculostomy tract hemorrhage, tube exposure due to inadequate tunneling). Patients who received revision operations due to technical skill-related issues were excluded, as shown in Fig. 1. The shunt revision rate comprised both the initial and total revision rates. The initial revision rate was the ratio of the total number of patients with at least one revision to the total number of patients included in this study. The total revision rate was the ratio of the total number of revisions to the total number of ventricular CSF shunt operations. Shunt valve replacement, external ventricular drainage, and distal shunting method conversion were the types of revision operations. Revision-free survival was defined as the length of time between index ventricular CSF shunt and the first revision operation.

Secondary outcome measures were to compare the shunt revision rate and revision-free survival between PVs and NPVs among different hydrocephalus etiologies.

### Statistics

The data were analyzed using IBM SPSS (version 22, IBM Corporation) and GraphPad Prism (version 8.0.0). Descriptive analysis for continuous variables and categorical variables was provided, including mean, standard deviation, and percentage. Comparative analysis regarding demographic data, index ventricular CSF shunt perioperative factors, revision rate, and revision cause between PV and NPV groups was done with an independent *t*-test for continuous variables and chi-square test and Fisher’s exact test for categorical variables. The revision-free survival was compared with the Kaplan-Meier curve using the log-rank test between PVs and NPVs among different hydrocephalus etiologies. Statistical significance was reached when the *p* value was less than 0.05.

## Results

### Patient characteristics

A total of 325 patients were included in the study, of which 181 patients receiving a PV placement (55.7%) and 144 patients receiving a NPV placement (44.3%). No significant statistical difference regarding demographic data and index ventricular CSF shunt factors was noted between two groups (Table 1). For the reason or indication for ventricular CSF shunts, there were more patients with cerebral aneurysmal SAH receiving PV than NPV (41.7%, 75/181 versus 21.5%, 31/144, *p* = 0.000). Otherwise, no statistical difference between the two groups was observed among all the other etiologies (Table 2). The most common indication for ventricular CSF shunt was nontraumatic intracranial hemorrhage in both groups (58.9% in the PV group and 49.3% in the NPV group). Interestingly, the second most common diagnosis requiring a ventricular CSF shunt differed in two groups, with iNPH (17.8%) in the PV group and intracranial neoplasm (19.4%) in the NPV group.

**Table 1:** Patient characteristics

	PV	NPV	*p* value
	*n* = 181 (55.7%)	*n* = 144 (44.3%)	
Age: mean, (years)	60.1 (15.4)	58.9 (16.8)	0.502
Gender: male	93 (51.1%)	85 (59.0%)	0.19
Gender: female	88 (48.9%)	59 (41.0%)	
Height (cm)	160.9 (8.2)	162.0 (8.3)	0.254
Weight (kg)	61.8 (12.2)	62.6 (14.9)	0.599
BMI	23.8 (3.8)	23.8 (5.1)	0.94
Medical disease			
Hypertension	108 (60.0%)	88 (61.1%)	0.929
Diabetes	42 (23.3%)	28 (19.5%)	0.478
Hyperlipidemia	16 (8.9%)	10 (6.9%)	0.664
Surgery parameter			
Operation duration (min)	88.3 (22.9)	86.2 (26.4)	0.445
Ventricular catheter laterality			
Right	136 (75.0%)	100 (69.4%)	0.266
Left	45 (25.0%)	44 (30.6%)	
Distal shunting site			
VPS	180 (99.4%)	142 (98.6%)	0.436
VAS	1 (0.6%)	2 (1.4%)	
Previous EVD	119 (66.1%)	99 (68.8%)	0.615
Postoperative antibiotics duration (days)	3.7 (4.8)	4.1 (4.2)	0.476

*BMI* body mass index, *VPS* ventriculo-peritoneal shunt, *VAS* ventriculo-atrial shunt

**Table 2:** Etiology for index CSF shunting surgeries

	No. of shunts (%)		
	PV	NPV	*p* value
	*n* = 181	*n* = 144	
Nontraumatic intracranial hemorrhage	106 (58.9)	71 (49.3)	0.085
Cerebral aneurysm SAH	75 (41.7)	31 (21.5)	0*
Hypertensive ICH	27 (15.0)	31 (21.5)	0.13
AVM	3 (1.7)	5 (3.5)	0.298
Spontaneous IVH	1 (0.6)	4 (2.8)	0.107
Intracranial neoplasm	22 (12.2)	28 (19.4)	0.074
Idiopathic normal pressure hydrocephalus	32 (17.8)	18 (12.5)	0.191
Trauma	13 (7.2)	19 (13.2)	0.073
Ischemic stroke	4 (2.2)	3 (2.1)	0.932
CNS infection	1 (0.6)	3 (2.1)	0.216
Others*	3 (1.1)	2 (1.4)	0.822

^*^The etiology “others” included in our study refers to 2 arteriovenous dural fistula patients with PV, 1 Wilson’s disease patient with PV, and 2 idiopathic intracranial hypertension patients with NPV. We group them into “others” due to the rarity of these diseases

### Shunt revisions

With a minimum follow-up of 5 years, there were 23 patients (12.8%) with PV and 22 patients (15.3%) with NPV receiving an initial revision operation (Table 3). No significant statistical difference in the initial revision rate was seen between two groups (*p* = 0.52). In both groups, more than 50% of patients with an initial revision operation underwent further revision operations. There were 52 revision operations in the PV group and 42 in the NPV group. No significant statistical difference in the total revision rate was found between two groups (22.4% versus 22.6%, *p* = 0.968). More than 50% of revision operations were performed within the first year in both groups (78.3% in PV; 86.4% in NPV). Shunt revision-free survival for the patients receiving revision operations in the PV and NPV groups was 91 days and 62.5 days, respectively.

**Table 3:** Ventricular CSF shunt operation and revision operation times

	PV	NPV	*p* value
Total no. of index shunts	181	144	
Total no. of ventricular CSF shunts	233	186	
Patients with initial revision	23 (12.8%)	22 (15.3%)	0.52
Total revision times	52 (22.4%)	42 (22.6%)	0.968
Revision times			
1	9	10	
2	8	6	
3	2	5	
4	0	0	
5	3	1	
6	1	0	
Patients with initial revision within 1 year	18 (78.3%)	19 (86.4%)	
Patients with initial revision more than 1 year	5 (21.7%)	3 (13.6%)	
Shunt revision-free survival, days	91	62.5	

### The cause of revision

The causes of initial revision and total revision were listed in Tables 4 and 5. There were more patients receiving an initial revision operation in the NPV group due to valve malfunction compared to the PV group (13 versus 3, *p* = 0.001). Of note, 9 patients in the NPV group and 2 patients in the PV group suffered from subdural hematoma due to over-drainage (*p* = 0.012). By contrast, for those who received an initial revision, shunt obstruction was more commonly seen in patients with PV than with NPV (*p* = 0.017). There was no statistical difference in infection as a cause of the initial revision between the two groups. Similar results were found for the cause of total revision operations.

**Table 4:** Cause of the initial revision

	PV	NPV	*p* value
Valve function-related	3	13	0.001*
Overshunting	3	10	0.016*
Subdural effusion/SDH noted	2	9	0.012*
Undershunting	0	3	0.067
Obstruction	12	4	0.017*
Reservoir obstruction	4	2	0.413
Proximal tip obstruction	2	0	0.157
Distal tip obstruction	6	2	0.136
Infection	8	5	0.250
CNS infection	5	4	0.766
Abdominal infection	3	1	0.317

The asterisk and the bold emphasis refer to *p* value which is smaller than 0.05

*SDH* subdural hemorrhage, *CNS* central nervous system

**Table 5:** Cause of the total revisions

	PV	NPV	*p* value
Valve function-related	4	16	0*
Overshunting	3	12	0.003*
Subdural effusion/SDH noted	2	10	0.004*
Undershunting	1	4	0.119
Obstruction	16	6	0.022*
Reservoir obstruction	5	2	0.23
Proximal tip obstruction	3	0	0.076
Distal tip obstruction	8	4	0.301
Infection	12	6	0.175
CNS infection	9	5	0.348
Abdominal infection	3	1	0.369
	32^#^	28^#^	

The asterisk and the bold emphasis refer to *p* value which is smaller than 0.05

^#^The total counts of the causes of revision are less than the total revision times due to multiple revisions from the same cause

### The impact of hydrocephalus etiology on revision rate

The initial shunt revision rates based on the hydrocephalus etiology in both groups were listed in Table 6. In iNPH and post-traumatic hydrocephalus patients, the revision rates were significantly lower in the PV group than in the NPV group (iNPH: 3.13% versus 22.2%, *p* = 0.031; Trauma: 0% versus 36.8%, *p* = 0.013). In nontraumatic intracranial hemorrhage patients, there was a trend that patients with PV more likely needed a revision operation than those with NPV (16% versus 7%, *p* = 0.075). No significant statistical difference in the revision rates was found among all the other etiologies.

**Table 6:** Initial shunt revision rates with hydrocephalus etiology and valve type

Etiology	PV (%)	NPV (%)	*p* value	HR
Nontraumatic intracranial hemorrhage (*n* = 177)	17/106 (16)	5/71 (7)	0.075	**2.38 (1.02–5.56)**
Cerebral aneurysm SAH (*n* = 106)	11/75 (14.7)	3/31 (9.7)	0.49	1.40 (0.50–3.95)
Hypertensive ICH (*n* = 58)	6/27 (22.2)	2/31 (6.5)	0.082	3.70 (0.92–14.9)
AVM (*n* = 8)	0/3 (0)	0/5 (0)	N/A	N/A
Spontaneous IVH (*n* = 5)	0/1 (0)	0/4 (0)	N/A	N/A
Intracranial neoplasm (*n* = 50)	5/22 (22.7)	5/28 (17.9)	0.669	1.33 (0.38–4.64)
iNPH (*n* = 50)	1/32 (3.1)	4/18 (22.2)	0.031*	**0.127 (0.02–0.81)**
Trauma (*n* = 32)	0/13 (0)	7/19 (36.8)	0.013*	N/A
Ischemic stroke (*n* = 7)	0/4 (0)	0/3 (0)	N/A	N/A
CNS Infection (*n* = 4)	0/1 (0)	1/3 (33)	1	0 (NA)
Others (*n* = 4)	0/3 (0)	0/2 (0)	N/A	N/A

The asterisk and the bold emphasis refer to *p* value which is smaller than 0.05

**Fig. 2:**
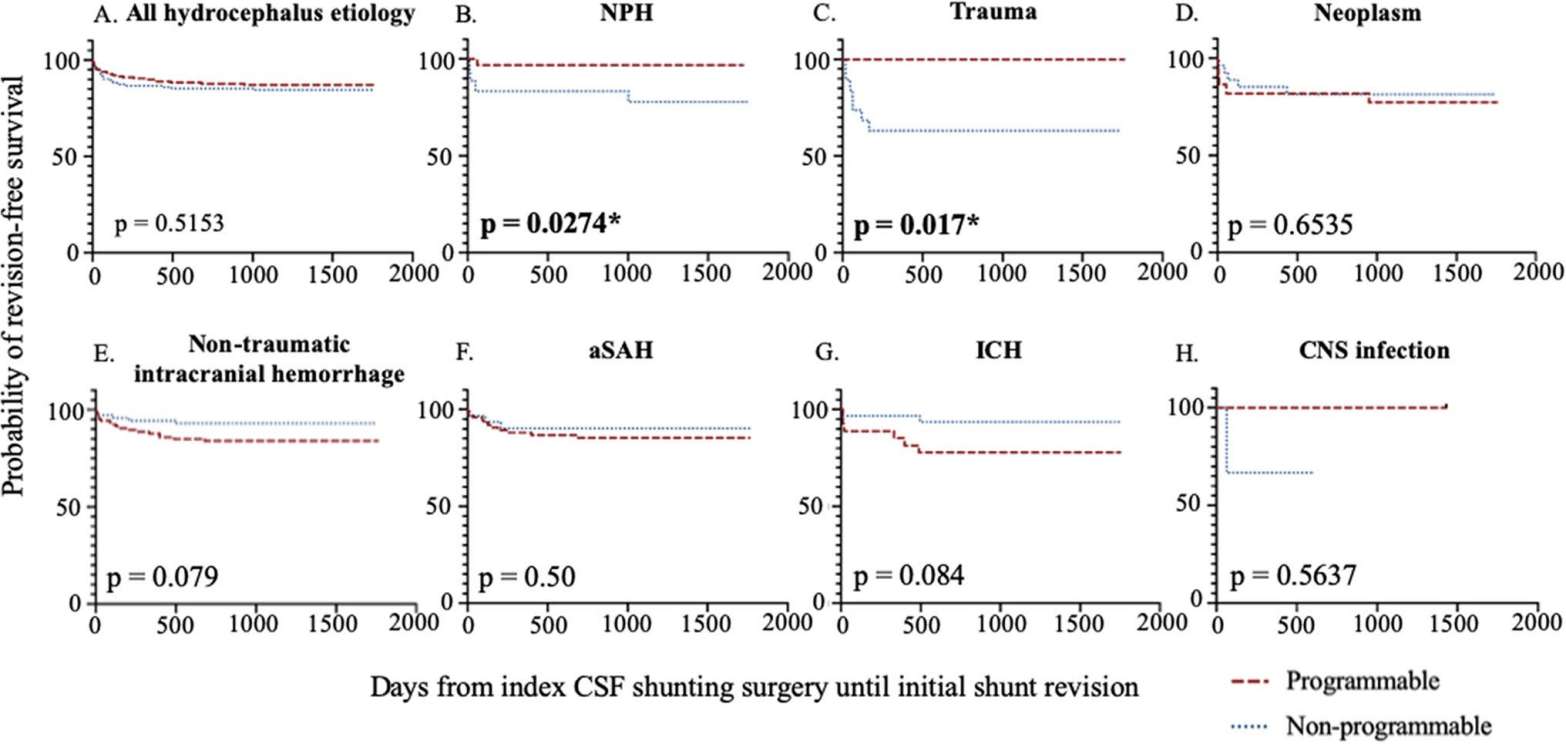
Revision-free survival curve between PVs and NPVs among different hydrocephalus etiologies. Probability of shunt revision-free survival with time in days was estimated by Kaplan-Meier survival curve analysis to compare PVs and NPVs among different hydrocephalus etiologies. *p* value calculated with log-rank test was labeled at the left bottom corner of each figure. No revision-free survival difference was found between PV and NPVs among all hydrocephalus etiologies (**A**). Revision-free survival benefit of PV compared to NPV was noted in iNPH and post-traumatic hydrocephalus patients (**B**, **C**). A trend of revision-free survival benefit of NPV compared to PV, which was not statistically significant, was noted among nontraumatic intracranial hemorrhage patients including aSAH and hypertensive ICH patients (**E**–**G**). No revision-free survival difference was noted among all the other hydrocephalus etiologies (**D**, **H**)

The survival curves for different hydrocephalus etiologies are shown in Fig. 2. No survival difference was seen between the PV and NPV groups. Similar to the results of the initial revision rate, revision-free survival benefit was observed in the PV group for iNPH (*p* = 0.0274) and post-traumatic hydrocephalus patients (*p* = 0.017). Despite no statistical significance, for nontraumatic intracranial hemorrhage patients, there was a trend that the NPV group was more likely to have a revision-free survival benefit compared to the PV group (*p* = 0.079). Otherwise, no revision-free survival difference was noted among all the other etiologies.

## Discussion

This is the first study to the best of our knowledge to follow up every shunted adult patient with hydrocephalus for at least a 5-year-period to compare the efficacy and durability of PV to NPV. Mixed results have been yielded by multiple previous studies due to the complexity of cause of the revision operation and the heterogenous etiology in adult hydrocephalus [1, 22, 28, 33, 39]. Our result suggested no difference in the initial revision rates, total revision rate, and shunt revision-free survival between the PV and NPV groups among all adult patients with hydrocephalus. By contrast, the difference in the revision rate and revision-free survival between the PV and NPV group was identified in our study among patients with specific hydrocephalus etiologies. We hypothesize that the difference in the durability of the PV and NPV in the setting of different hydrocephalus etiologies is associated with the interplay between the clinical nature of each hydrocephalus etiology and the specifications and features of PV/NPV.

### Features of PV and NPV

We found that NPVs were more susceptible to under-drainage or over-drainage compared to PVs in accordance with the previous studies [21, 33]. The opening pressure of NPVs cannot be adjusted when patients encounter neurological symptoms secondary to either under-drainage or over-drainage. The only solution is to perform a revision operation, and surgical evacuation is sometimes required for over-drainage-induced subdural hematoma/effusion. By comparison, the adjustability of PVs is an advantage that neurosurgeons can timely change the opening pressure in response to under-drainage or over-drainage, which could also prevent a revision or the formation of subdural hemorrhage/effusion. Moreover, it has been shown that the early fine adjustments of PVs within the first 6 months after implantation could prevent over-drainage or under-drainage [30].

Interestingly, a higher obstruction rate within the programmable ventricular CSF shunts compared to NPVs was identified in our study, which was first noted among previous studies. Multiple previous studies compared shunt obstruction rates between patients with PV and NPV, and the majority of them revealed a similar obstruction rate in both pediatric and adult patients [1, 22, 29]. The obstruction within the ventricular CSF shunting system could develop in the proximal catheter, reservoir, or distal catheter [9, 28]. The shunting system of PV and NPV shares the same characteristics such as the inner diameter of the ventricular tube and the peritoneal tube, as well as the size of the side hole of the ventricular tube. The major difference of PVs and NPVs is the presence of magnetic-assisted adjustable pressure valve. Christoph et al. revealed that 8 explanted programmable valves taken from the adult hydrocephalus patients with shunt malfunction showed significant flow rate change differing from the manufacturer’s suggestion in vitro [4].

We hypothesize that the more delicate interior design of the programmable valves might play an important role in the development of obstruction and malfunction. Further in vitro and in vivo studies are needed to investigate and validate the higher obstruction rates among the programmable valves compared to NPVs.

### The clinical nature of different hydrocephalus etiologies

Previous studies have revealed that the etiology of hydrocephalus plays an important role in the ventriculo-peritoneal shunt (VPS) revision rates [1, 27, 32]. In iNPH patients, the relatively thin and clear consistency of the CSF leads to the low obstruction rate in both PV and NPV groups. Most of the shunted iNPH patients require a revision operation due to either over-drainage or under-drainage [28, 33]. The use of PV in iNPH patients could help neurosurgeons adjust the opening pressure timely to prevent a revision operation and reach a better revision-free survival. Patients with acute traumatic brain injury, even mild head trauma, could suffer from delayed onset subdural hemorrhage or effusion due to the disruption of the meningeal blood–brain barrier and the injury of cortico-dural bridging vessels [3, 6, 13, 35]. Besides, it has been shown that the cortico-dural bridging vessels were at higher risk of rupture after ventricular shunting [16]. In our study, most post-traumatic hydrocephalus patients with NPV received a revision due to over-drainage and over-drainage–induced subdural effusions, and we found the PV in the post-traumatic hydrocephalus patients had a better shunt revision-free survival and a lower revision rate when compared to the NPV. We hypothesize that the disrupted dura-arachnoid connection and potential bridging vessel injury due to acute trauma might greatly raise the risk of over-drainage-induced subdural hematoma in the presence of a ventricular shunt, leading to worse revision-free survival in the NPV group.

Nontraumatic subarachnoid hemorrhagic hydrocephalus patients with ventricular CSF shunts have been shown with higher risks to receive revision operation compared to iNPH [32]. In vitro studies have shown that the hemorrhagic CSF would impair the shunt performance due to the partial or complete blockage of the valve [5].Though not significant, there is a trend (*p* = 0.075) that NPV might be superior to PV for nontraumatic intracranial hemorrhage patients in our study. To date, only three studies compared the efficacy and safety between PVs and NPVs in adult patients with nontraumatic subarachnoid hemorrhagic hydrocephalus. Two studies reported that PVs were related to a lower revision and obstruction rate [20, 28], whereas Darkwah et al. showed no difference in valve dysfunction between PVs and NPVs [9]. The discrepancy between our study and previous studies could be due to the following: (1) The mismatch of sample size between PV and NPV groups was significant, which led to the statistical bias. (2) The mean follow-up time was not long enough for complications/obstruction to develop. Further investigation with in vivo studies with larger sample size and longer follow-up period is needed to evaluate if programmable valves are more susceptible to obstruction in patients with bloody CSF, for example, aneurysmal SAH patients when compared to NPVs.

As for the other etiologies, such as brain neoplasm, ischemic stroke, central nervous system (CNS) infection, no difference in the initial revision rate, total revision rate, and revision-free survival between PV and NPV groups was observed. Tumor-related hydrocephalus patients with ventricular CSF shunts were reported to have a shunt failure rate around 33% [15, 32], which is in accordance with our results. Sex, age, tumor location, previous EVD placement, previous craniotomy, post-craniotomy hemorrhage, and post-craniotomy meningitis were found as non-significant risk factors for revision in adult brain tumor patients [15]. Further studies are needed to clarify the underlying cause of relatively high shunt failure rate in adult brain tumor patients with either PVs or NPVs. For the rare onset of ischemic stroke and CNS infection–related hydrocephalus, it warrants a nationwide or multi-center study to recruit more patients to compare the efficacy and durability between PV and NPVs.

### Limitations

Several limitations are present in our study. First, the nature of a retrospective study limited the analysis in a randomized-controlled way. The patient number of PV group and NPV group differs among different hydrocephalus etiologies due to the choice between PV and NPV based on the attending neurosurgeons’ preferences in our institution. The patient’s economical affordability is also an important consideration for attending neurosurgeon when choosing between PV or NPV. PV had not been covered by Taiwan National Health Insurance until July 2022. NPV might be a more affordable treatment for certain hydrocephalus patients such as tumor patients while there is no strong evidence to support the benefit of PV use. Therefore, a biased interpretation of the statistical results may exist. However, the possible bias originating from the patient number difference in PV and NPV is statistically acceptable among all hydrocephalus etiologies except aSAH. For example, there is a trend that “PV use is higher in iNPH patients” and “NPV use is higher in tumor patients” in our study, but the number of PV or NPV use in both iNPH and tumor patients is not statistically significantly different (iNPH: *p* = 0.191; tumor: *p* = 0.074). Besides, the small patient number of several subgroups in both PV and NPV including AVM, spontaneous IVH, ischemic stroke, and others may not lead to a statistically significant conclusion. It is possible for these patients to develop hydrocephalus with the CSF shunting needs, but the incidence is relatively low. For example, previous studies revealed that ischemic stroke patient accounts for less than 1%, and CNS infection patient accounts for only 1.3% among adult hydrocephalus patients [11, 24]. Despite the rarity of these diseases, it remains an unsolved medical problem regarding the efficacy of PV and NPV use among them. Second, the difference in proficiency levels in surgical techniques and decision making among different neurosurgeons and in the same neurosurgeon at different training stages might have an impact on the revision occurrence though the technical skill-related revision has been excluded from our study. Finally, the complexity of reasons leading to revision operations in the same patient complicated the analysis of risk factors/causes. Further randomized-controlled trials or prospective studies for each specific hydrocephalus etiology are needed to clarify the indications of PV and NPV implantation.

## Conclusions

The combination of the different etiologies of hydrocephalus and the features of PV and NPV results in different outcomes—revision rate and revision-free survival. The present study is the first to reveal that PV could reduce the revision rate and improve patient’s quality of life among those with post-traumatic hydrocephalus compared to NPV, which is objective evidence for neurosurgeons when choosing PV versus NPV. The present study also reveals the PV use might be superior to NPV use in iNPH patients, which is in accordance with previous studies. Further studies are needed to define the indications of PV and NPV use in adult patients among different hydrocephalus etiologies such as nontraumatic intracranial hemorrhage.

## Data Availability

All de-identified datas are archived and available under request with CGMH institutional review board permission.

## Abbreviations

aSAH: Aneurysmal subarachnoid hemorrhage
AVM: Arteriovenous malformation
BMI: Body mass index
CNS: Central nervous system
CSF: Cerebrospinal fluid
EVD: External ventricular drainage
ICH: Intracerebral hemorrhage
iNPH: Idiopathic normal pressure hydrocephalus
NPV: Non-programmable valve
PV: Programmable
VPS: Ventriculo-peritoneal shunt
